# Factors associated with inappropriate urine culture orders in hospitalized patients with indwelling urinary catheters

**DOI:** 10.1017/ash.2024.466

**Published:** 2024-12-30

**Authors:** Ramez Azzam, Nicole C. Nicolsen, Jacob W. Pierce

**Affiliations:** 1 Brody School of Medicine at East Carolina University, Greenville, NC, USA; 2 ECU Health System, Greenville, NC, USA

## Abstract

Urine cultures ordered for patients with indwelling urinary catheters might lead to reporting of non-clinically significant catheter-associated urinary tract infections (CAUTIs) or asymptomatic bacteriuria to the National Healthcare Safety Network (NHSN). We examined factors associated with inappropriate urine cultures orders leading to reporting of non-clinically significant CAUTIs to NHSN.

## Introduction

Catheter-associated urinary tract infections (CAUTIs) are among the most prevalent healthcare-associated infections.^1^ Clinical diagnosis of CAUTIs and National Healthcare Safety Network (NHSN) definitions do not always align.^2^ Most patients with indwelling urinary catheters develop asymptomatic bacteriuria (ASB) caused by bacterial colonization and may be misdiagnosed as CAUTIs.^3^ This ultimately leads to inappropriate reporting to NHSN and often unnecessary antimicrobial use. In this study, patients with urinary catheters with reported CAUTIs were examined aiming to define factors related to inappropriate urine culture ordering leading to reporting of non-clinically significant positive urine cultures to NHSN.

## Materials and methods

This study was conducted at a 973-bed tertiary care academic medical center in Eastern North Carolina. All NHSN-reported CAUTIs were evaluated from October 2021 until July 2023 by reviewing electronic medical records. The CAUTIs were identified based on NHSN definitions: patient with indwelling urinary catheter that have been in place for more than 2 consecutive days, with the urinary catheter present for any portion of the calendar day on the date of diagnosis of CAUTI or removed the day before the date of CAUTI diagnosis; along with presence of any of the following signs or symptoms: fever (>38.0°C), suprapubic tenderness, costovertebral angle pain or tenderness, urinary urgency, urinary frequency, or dysuria. The definition also excludes urine cultures with more than two species of organisms identified, at least one of which is a bacterium of ≥10^5^ CFU/mL; and excludes urine cultures positive for yeast, mold, or parasite.^4^

The data collected included: age, sex, time of urine culture order (dayshift 7:00 am–5:59 pm versus nightshift 6:00 pm–6:59 am), provider type (attending physician, Advanced Practice Provider or resident), white cell count in urine analysis, presence of infectious disease consultation, if urine culture was performed from a urinary catheter placed ≤ 7 days ago (labeled insertion) or from urinary catheter placed > 7 days ago (labeled maintenance), and days that the urinary catheter was in place (date of initial placement or date of last exchange if an exchange was performed). A logistic regression model was fit for patients treated for CAUTI. Inappropriate urine culture was defined as a positive urine culture for microorganisms, as determined by NHSN CAUTI definition, which did not prompt antimicrobial treatment. All data analysis was performed using SAS (SAS 9.4, Cary, NC: SAS Institute Inc., 2002–2023). Ethical approval for this study was evaluated by the ECU Institutional Review Board (IRB) and approved as IRB exempt.

## Results

Table 1 demonstrates patient characteristics stratified by treatment for CAUTI. The number of NHSN-reported CAUTIs that were not actually treated was 22 out of 116 (19%). The study included 68 males and 48 females. The mean age of patients treated for CAUTI was 62 years, while the average age of patients not treated for CAUTI was 54.7 years. The total number of dayshift urine culture orders was 91, of which 78 (86%) prompted treatment for CAUTI. The nightshift urine culture order total was 25, of which 16 (64%) prompted treatment for CAUTI. The analysis suggests that positive urine cultures ordered during nightshift were less likely to be treated with antibiotics, and this result was statistically significant in both the adjusted (OR 0.21; p-value 0.01) and unadjusted (OR 0.30; p-value 0.02) analyses—see Table 2. The model also suggests that positive urine cultures ordered by residents and for older patients were more likely to be treated for CAUTI, but these results were not statistically significant—see Table 3. Finally, the longer a catheter was in place the less likely a positive urine culture was to be treated (OR 0.89; p-value 0.003)—see Table 3.

**Table 1.** Patient characteristics stratified by treatment for CAUTI

	Treated for CAUTI (n = 94)	Not Treated for CAUTI (n = 22)	Total Number
Male Sex (n,%)	53 (56.4%)	15 (68.2%)	68
Female Sex (n, %)	41 (43.6%)	7 (31.8%)	48
Mean Age	62 (±16.2)	54.7 (±21.1)	–
Mean Days Catheter in Place	7 (±5.2)	13.9 (±16.2)	–
Dayshift Urine Culture Orders (n, %)	78 (83%)	13 (59.1%)	91
Nightshift Urine Culture Orders (n, %)	16 (17%)	9 (40.9%)	25
Attending Ordered (n, %)	21 (22.3%)	5 (22.7%)	26
Advanced Practice Provider Ordered (n, %)	33 (35.1%)	12 (54.6%)	45
Resident Ordered (n, %)	40 (42.6%)	5 (22.7%)	45
Insertion (n, %)	44 (46.8%)	8 (36.4%)	52
Maintenance (n, %)	50 (53.2%)	14 (63.6%)	64
ID Consulted (n, %)	8 (8.5%)	7 (31.8%)	15
ID Not Consulted (n, %)	86 (91.5%)	15 (68.2%)	101

**Table 2.** Unadjusted logistic regression model for time of day of urine culture order (dayshift 7:00 am–5:59 pm, nightshift 6:00 pm–6:59 am) for **likelihood** of treatment for CAUTI

	OR	95% CI	*P* value
Nightshift Urine Culture Orders	0.30	0.11–0.81	0.02*

**Table 3.** Results of adjusted logistic regression model

	OR	95% CI	*P* value
Nightshift Urine Culture Orders	0.21	0.061–0.74	0.01*
Advanced Practice Provider Ordered	0.73	0.19–2.8	0.65
Resident Ordered	1.4	0.31–6.6	0.65
Male Sex	0.65	0.21–2.1	0.47
Mean Age	1.03	1.0–1.06	0.05
Days Catheter in Place	0.89	0.83–0.96	0.003*

## Discussion

It is estimated that one million cases of CAUTI are reported per year in United States hospitals and nursing homes (1). However, this number might be overestimated as many ASBs might be misinterpreted as CAUTIs. In actual clinical practice diagnosing a CAUTI is much more complicated, as many patients with indwelling urinary catheter and ASB might be reported as a CAUTI based on NHSN definitions (4). In this study, we found that some CAUTIs reported to NHSN did not prompt antibiotic prescription and therefore could be classified as inappropriate urine culture orders. A limitation of this study is that some patients treated with antibiotics may not have had clinically significant urinary tract infections. However, making this clinical determination can be subjective, and we elected for a pragmatic approach that likely errs on the side of over-calling the clinical significance of positive urine cultures. As shown in Table 2, positive urine cultures ordered during nightshifts were less likely to be treated with antibiotics. This result was statistically significant in both the adjusted and unadjusted analyses as shown in the results section above. Per Tambyahe et al., more than 90% of CAUTI diagnoses are asymptomatic and rarely cause bloodstream infections.^5^ Therefore, efforts should be aimed at reducing the numbers of urine culture orders in patients with indwelling urinary catheters especially at night. It is unclear why this correlation exists; however, it might be attributed to the lower number of staff working at night with the same number of patients, leading to less time to independently evaluate patients for clinical symptoms. Moreover, Advanced Practice Providers and residents have less direct supervision by attending providers at night possibly leading to inappropriate urine cultures performed in patients without clear urinary symptoms. Automated electronic medical record based clinical decision support tools may be an effective tool to guide providers regardless of time of day. Additional research is needed to determine the most effective strategies for improving diagnostic stewardship in nightshift providers.

## Conclusion

Positive urine cultures that did not prompt antimicrobial treatment could be considered inappropriate urine culture orders that resulted in over-reporting of CAUTIs to NHSN. Positive urine cultures ordered by the nightshift team were less likely to be treated, and this phenomenon may be an important target for diagnostic stewardship interventions to reduce inappropriate urine culture orders and over-reporting of CAUTIs.
